# Fluorescent proteins: A journey from the cell to extreme environments in material science

**DOI:** 10.1111/php.70011

**Published:** 2025-07-21

**Authors:** Bianca Menchicchi, Andre C. Stiel, Mattia Nieddu, J. P. Fuenzalida‐Werner

**Affiliations:** ^1^ BIOMA Institute for Biodiversity and Environment, University of Navarra Pamplona Spain; ^2^ Chemistry Department, School of Science University of Navarra Pamplona Spain; ^3^ Institute of Biological and Medical Imaging, Bioengineering Center, Helmholtz Zentrum München Neuherberg Germany; ^4^ Chair of Biological Imaging, Central Institute for Translational Cancer Research (TranslaTUM), School of Medicine and Health & School of Computation, Information and Technology, Technical University of Munich Munich Germany; ^5^ Protein Engineering for Superresolution Microscopy Lab, University of Regensburg Regensburg Germany; ^6^ Molecular Nanotechnology Lab, Organic Chemistry Department University of Alicante Alicante Spain

**Keywords:** circularly polarized luminescence (CPL), fluorescent proteins, imaging and spectroscopy, polysaccharides and protein interactions, protein kinetic stability, protein stabilization strategies, supramolecular materials

## Abstract

This review presents the progression from the use of fluorescent proteins (FPs) and chromoproteins as bioimaging labels and sensors to the strategic engineering of their properties for robust functionality in synthetic and non‐biological environments. Specifically, engineered variants of the small ultra‐red fluorescent protein (smURFP) were developed and optimized for optoacoustic imaging through structure‐guided mutagenesis. Reversibly switchable genetically encoded indicators were also created to enhance bioimaging capabilities. To extend the applicability of such proteins to material science and enable their function in everyday applications—such as environmental sensors, encoders, or color components in textiles and electronics—their inherent stability limitations were addressed. For this purpose, supramolecular stabilization strategies, including genetically encoded macro‐oligomerization techniques, were explored. These methods effectively enhanced the resilience of FPs under chemically challenging conditions, without compromising their photophysical properties. Finally, the exploration of circularly polarized luminescence (CPL) from FPs is discussed, and their potential as CPL emitters suitable for sustainable photonic applications is identified. Overall, the transformative potential of engineered FPs as essential components for applications beyond bioimaging is emphasized.

## Graphical abstract

Applications of proteins and microorganisms as tools or effectors in material science.

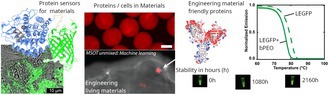

**Abbreviations**
aFt: Apoferritin
BCPL: Circularly Polarized Luminescence Brightness
BFP: Blue Fluorescent Protein
BV: Biliverdin
CAP: Chitosan Affinity Protein
CD: Circular Dichroism
CPL: Circularly Polarized Luminescence
DCM: Dichloromethane
DLS: Dynamic Light Scattering
eGFP: Enhanced Green Fluorescent Protein
FPs: Fluorescent Proteins
FRET: Förster Resonance Energy Transfer
GCaMP: Genetically Encoded Calcium Indicator
glum: Luminescence Dissymmetry Factor
HPC: Hydroxypropyl Cellulose
HT: Hula Twist
IC: Internal Conversion
ISC: Intersystem Crossing
LEGFP: Leucine Zipper eGFP Construct
mGL: mGreenLantern
MLS: Multimodal Laser Spectrometer
MSOT: Multispectral Optoacoustic Tomography
OA: Optoacoustic
PBS: Phosphate‐Buffered Saline
PDB: Protein Data Bank
PEO: Polyethylene Oxide
PMMA: Polymethyl Methacrylate
RSFPs: Reversibly Switchable Fluorescent Proteins
rsGEIs: Reversibly Switchable Genetically Encoded Indicators
scmGL: Supercharged mGreenLantern
sfGFP: Superfolder Green Fluorescent Protein
smURFP: Small Ultra‐Red Fluorescent Protein
Tm: Melting Temperature
Tnr: Temperature of Non‐Reversibility
VR: Vibrational Relaxation

## INTRODUCTION

In everyday life, we encounter countless colorful materials that enhance our quality of life, from packaging and optics to sensors, coatings, paints, and electronics. However, since the 19th century, we have relied mainly on polymers and colorants derived from petrochemicals or inorganic compounds.^1^, ^2^, ^3^, ^4^ Critically, these materials have become a persistent source of environmental pollution because they are often designed for single‐use applications. As a result, the sustainable development of efficient, colorful, and emissive materials is inseparably tied to the vision of a modern, green lifestyle.^1^, ^2^, ^3^ Moreover, the boundaries inherent to synthetic chemistry impose limitations on the tuning of photochromic properties, while fluorescent proteins (FPs) can benefit from both random and directed evolution strategies,^5^ a flexibility unavailable to synthetic dyes.The inherent drawbacks of synthetic colorants or dyes, in terms of sustainability and functionality, can be overcome by using chromo‐ or fluorescent proteins embedded in biopolymer matrices. FPs have profoundly impacted scientific research by enabling visualization and tracking of biological processes at the molecular and cellular levels as labels and sensors. Notably, only a few years after the cloning and engineering of green fluorescent protein (GFP) began, FPs research took early steps into materials science, FPs being incorporated into synthetic matrices for in vitro studies via confocal microscopy or as a test sample for new imaging techniques.^6^, ^7^ The trend has only accelerated since then, with engineered FPs serving as innovative functional components.^8^, ^9^ This expansion is mainly because FPs, as proteins, are inherently biocompatible, and due to their genetic encoding, can be mass‐produced in easily cultivable hosts such as the bacterium *Escherichia coli* (*E. coli*). Beyond that, the protein surrounding the chromophore offers the possibility for elaborate tuning of photophysical properties beyond what individual dye molecules could accomplish.

This review highlights the route from developing FP‐based labels and sensors to genetically engineering these proteins to enhance their stability and functionality for materials science applications. We illustrate this evolution with examples from our different published works discussed in the context of current literature. Our research extends beyond bioimaging and explores how FPs can be adapted for their use in complex and non‐native environments. This includes their integration into synthetic polymers, supramolecular assemblies, and living materials, all while preserving or enhancing their photophysical properties. By combining structural biology, protein design, and materials science, we demonstrate how these proteins can be repurposed from imaging tools to tunable components for sustainable materials and optoelectronic devices. This progression—from fundamental biology to applied material science—illustrates the growing potential of FPs as key players in the development of future bio‐based materials.

## BIOIMAGING—FROM PROTEIN STRUCTURE TO TAILORED PHOTOPHYSICS

Structural biology, spectroscopy, and protein engineering were combined under the lead of Professor Dr. Andre C. Stiel for the design of labels and sensors for optical imaging.

**FIGURE 1:**
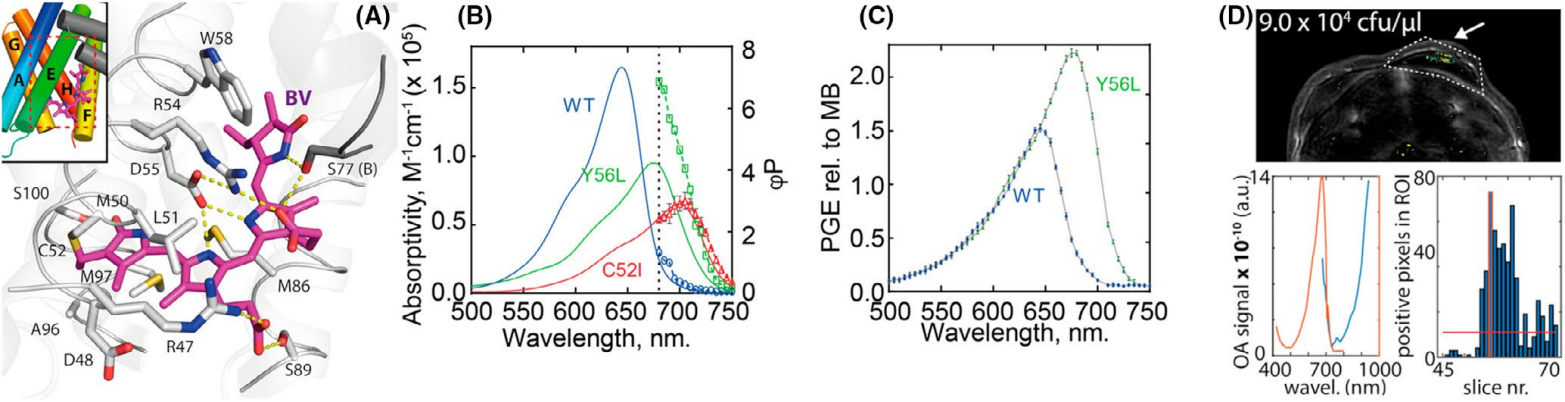
(A) Crystal structure of a smURFP‐Y56R mutant covalently bound to the biliverdin (BV) chromophore (PDB entry 6FZN). (B) Absorption spectra and MSOT‐derived OA molar efficiency of wild‐type smURFP and mutants C52I and Y56L, which are non‐covalently and covalently bound to BV, respectively. (C) OA spectra of wild‐type smURFP and variant Y56L measured with a custom‐built OA spectrometer. (D) In vivo MSOT imaging of *E. coli* expressing smURFP‐Y56L embedded in Matrigel after injection in the back of a Fox‐N1 nude mouse. Figures modified from refs [10, 11].

The fluorescent phycobiliprotein smURFP (small Ultra‐Red Fluorescent Protein) was initially engineered to enhance its utility for Multispectral Optoacoustic Tomography (MSOT).^10^, ^11^ This work illustrates that tailored design, grounded in direct optoacoustic (OA) measurements and a deep understanding of protein structure and photophysical relationships, is essential for developing the next generation of imaging agents. In the OA phenomenon, the absorbed light is converted into ultrasound waves due to the thermoelastic expansion following the non‐radiative decay. Thus, guided by structural insights into the chromophore‐binding site (Figure 1A), mutations were introduced in smURFP to promote non‐radiative decay and red‐shift absorption further towards near‐infrared wavelengths, making the protein more suitable for deep‐tissue imaging. Variants with reduced fluorescence quantum yields—and consequently significantly increased optoacoustic (OA) signal strength—were generated by replacing polar residues in the chromophore pocket with hydrophobic and aromatic amino acids (Figure 1B). Furthermore, mutants of smURFP bind biliverdin (BV) noncovalently, such as C52I and exhibited red‐shifted absorption and enhanced OA performance due to an increased conjugation of the BV chromophore and its lack of stabilization. Similarly, the covalent variant Y56L showed improved OA properties, most likely as a consequence of changes in binding pocket polarity and chromophore planarity (Figure 1C,D). Others have postulated a similar redshift as a consequence of changes in the binding pocket in the context of phycobiliprotein photophysics.^12^, ^13^ Our study demonstrates the feasibility of structure‐guided mutagenesis to re‐engineer fluorescent proteins for non‐fluorescent, OA‐specific applications.

**FIGURE 2:**
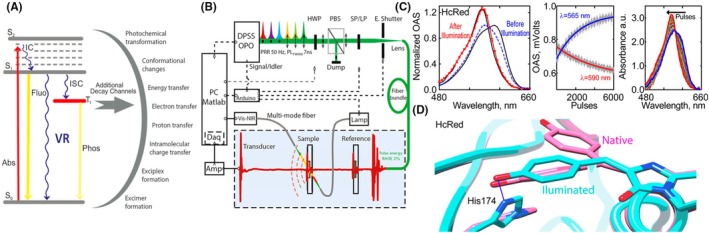
(A) Photophysics of OA signal generation and common competing decay channels. Abs: absorbance, S0: ground state, S1: singlet excited state, T1: triplet excited state, VR: vibrational relaxation, ISC: intersystem crossing, IC: internal conversion, Pho: phosphorescence, and Fluo: fluorescence. (B) Multimodal laser spectrometer diagram, for details see ref. [14] (C) Photoswitching effects in HcRed. (D) Structure of HcRed focusing on the chromophore. In blue: Structure of HcRed showing a *trans* chromophore after 60,000 pulses of 565 nm light (6Y1G), and in pink: Structure of native non‐illuminated HcRed showing a *cis* chromophore (1YZW). Figure modified from ref. [14].

Building on these findings, the assumption that optical absorption spectra directly predict OA signal, an assumption at the heart of most spectral unmixing strategies in OA imaging, was challenged. Using a custom‐built multimodal laser spectrometer (MLS),^14^ absorbance, fluorescence, and vibrational relaxation—as reported by OA spectra—were measured for various dyes and fluorescent proteins (Figure 2A,B). The data revealed substantial discrepancies between absorption and OA spectra across multiple imaging agents, including indocyanine green, 800CW, and fluorescent proteins such as sfGFP (superfolder green fluorescent protein) and tdTomato (Tandem dimer Tomato). Notably, the data indicated that most likely the aggregation states, exciton coupling in dyes, and contribution from isomeric forms or neutral state of protein chromophores contributed to enhanced OA signal generation.^14^ It was further demonstrated that previously unreported photoswitching behavior—enabled by the high light fluence used in OA imaging—significantly influences OA signal efficiency in proteins such as tdTomato and HcRed (Heractis crispa Red) (Figure 2C). These changes were validated through structural determination of the photoswitched protein (Figure 2D). The spectroscopic findings were utilized in imaging applications, showing that improvements in spectral unmixing in in vivo imaging studies of mice can be achieved by understanding the OA signal efficiency of different aggregated states of indocyanine green or by applying the photoswitching behavior of the widely used label tdTomato.

Finally, expanding on label design, a new class of biosensors termed “reversibly switchable genetically encoded indicators” (rsGEIs) was introduced in Stiel's lab. These constructs were designed to combine the functionality of traditional genetically encoded indicators—which change their signal upon binding of an analyte of interest—with the photoswitching capability of reversibly switchable fluorescent proteins (RSFPs).^15^ By engineering a calcium sensor based on the circularly permuted Green Fluorescent Protein‐Calmodulin‐M13 Peptide (GCaMP5G), rsGCaMP variants exhibiting Ca^2+^‐dependent photoswitching behavior were developed. In a proof‐of‐concept work, it was shown that the ligand‐binding dependent photoswitching concept can be used in super‐resolution fluorescence microscopy and OA imaging alike. Structural characterization of these sensors revealed a different chromophore stabilization compared to the parent non‐sensor rsEGFP2, which possibly explains their improved switching speed and reduced photofatigue. Beyond that, the chromophore isomerization upon photoswitching shows a rare hula‐twist (HT), possibly due to the interaction of the calcium‐binding moiety and the beta‐barrel. Importantly, it was demonstrated that the concept is generalizable, with prototype rsGEIs developed also for maltose and dopamine.

## PROTEIN ENGINEERING FOR MATERIAL SCIENCE

**FIGURE 3:**
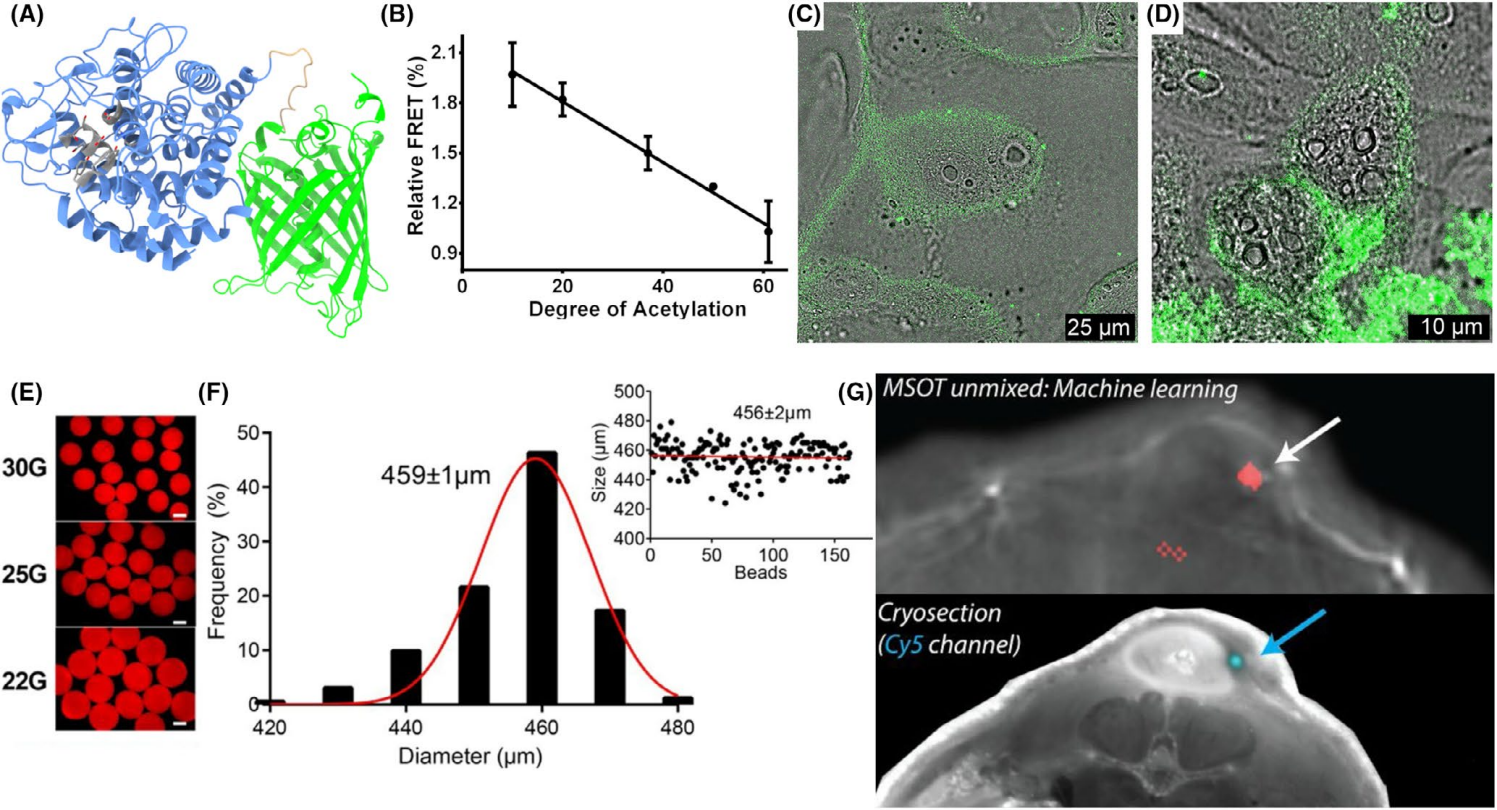
FRET chitin/chitosan sensor: (A) Structural model of CAP‐eGFP. (B) Correlation between the FRET behavior of CAP‐eBFP and CBP‐eGFP and the degree of acetylation of the substrates. (C) free chitosan labeled with CAP‐sfBFP and CAP‐eGFP. (D) chitosan‐tripolyphosphate (TPP) nanoparticles labeled with CAP‐sfBFP and CAP‐eGFP. Alginate beads (AlBes) as test samples: (E) Microscopy pictures of beads prepared with different gauge needles. (F) 30G beads diameter distribution with Gaussian fit probability density, with the insert showing an arithmetic average. (G) MSOT images of a 4T1 tumor model injected with an alginate bead loaded with *E. coli* expressing photo‐switching ReBphP‐PCM (arrow) and ground truth based on fluorescence imaging. Figure modified from refs [16, 18].

One modern example of fluorescent proteins as sensors in materials science can be traced to a sensor for chitin and chitosan, which was developed under the leadership of Professor Dr. Francisco Goycoolea and Dr. Stephan Kolkenbrock. In Goycoolea's lab, protein‐based tools exploiting Förster Resonance Energy Transfer (FRET) were used to track and characterize chitosan‐based materials. A key design strategy was implemented, involving the engineering of two types of fusion proteins.^16^, ^17^ The first, CAP (Chitosan Affinity Protein), was derived from a catalytically inactivated chitosanase with strong binding specificity for chitosan with a low degree of acetylation. The second, CBP (Chitin Binding Protein), was originated from a chitin‐binding domain that preferentially binds chitosan with a higher degree of acetylation. CAP and CBP were genetically fused to fluorescent proteins: blue fluorescent protein (BFP), namely enhanced BFP (eBFP) or superfolder BFP (sfBFP), as FRET donors, and green fluorescent proteins, namely eGFP or sf, GFP as FRET acceptors (Figure 3A). This setup allowed FRET efficiency to be measured as a function of the degree of acetylation, with the resulting signal intensity directly correlating with the acetylation level of the chitosan (Figure 3B). Compared to traditional techniques like ^1^H‐NMR (Proton nuclear magnetic resonance) or infrared spectroscopy, this method required much less material and instrumentation, making it accessible and efficient.

The CAP‐based FRET proteins adsorb efficiently onto the surface of the nanoparticles without destabilizing them, enabling noncovalent labeling. This labeling allowed real‐time monitoring of nanoparticle integrity, as the enzymatic degradation of the chitosan substrate by chitosanase resulted in a measurable loss of the FRET signal. Finally, the sensor was used to study how cells interact differently with chitosan polymers or nanoparticles. Confocal laser scanning microscopy revealed that labeled chitosan was distributed across the cell membrane (Figure 3C), whereas labeled chitosan nanoparticles were observed to accumulate as large and intensely fluorescent aggregates on the cell membrane (Figure 3D).

In another example of the approach to bridging protein science and materials science, the concept of engineering living materials with living mammalian cells or bacteria encapsulated in beads was used in Stiel's lab to meet the need for highly reproducible, physiologically relevant test samples.^18^ In this work, bacterial cells expressing photo‐switchable ReBphP‐PCM (Rhodopseudomonas palustris Bacteriophytochrome Photosensory Core Module) were encapsulated into alginate beads (AlBes). These FPs‐loaded beads, engineered as living test samples, were designed to mimic complex tissue features while allowing precise control over size, shape, and fluorophore/chromophore content, providing a robust system for validating optoacoustic imaging techniques (Figure 3E,F). The alginate matrix provided a non‐toxic and biocompatible scaffold for encapsulating living cells while maintaining their structural and optical integrity.

The AlBes were successfully employed as a tool for instrument calibration and benchmarking by serving as quasi point‐like sources for algorithm validation—especially for temporal unmixing strategies in complex biological environments—and for in vivo label performance comparison (Figure 3G). Importantly, the approach demonstrate the potential of fluorescent proteins (FPs) in material science and living materials, not only as contrast agents but also as functional components of engineered bio‐composite materials that support the development and refinement of emerging imaging technologies.

Despite being used in the study and characterization of materials, the FRET sensor and the FP‐rich bacteria encapsulated in the AlBes were limited to testing in an aqueous environment in order to preserve the integrity of the FPs and proteins. These biomolecules are generally constrained in nonbiological environments and lack resilience under unnatural stress conditions. Protein function is often lost when harsh conditions—such as exposure to organic solvents, high temperatures, high concentrations, or intense irradiation—are applied.

This problem was first encountered upon observing the low stability of enzymes outside their production microorganisms. Prior to employing protein engineering strategies, stabilization was first attempted through non‐covalent interactions. For example, in the lab of Professor Dr. Marc Ostermeier, a chimeric enzyme—the maltose‐activated β‐lactamase switch enzyme (MBP317‐347)—was studied and stabilized through its interaction with supramolecular assemblies composed of alginate, lysozyme, albumin, and lecithin. These assemblies were found to interact with the maltose‐binding pocket of the active (folded) conformation of MBP317‐347, promoting a stabilization supported by electrostatic interactions and hydrogen bonding.^19^ These types of interactions protected the enzyme from environmental stress, such as pH shifts and changes in ionic strength, substantially increasing enzyme stability, catalytic activity, and half‐life at 37°C from 2 to 37 h. Effectively, by this approach, the protein was “locked” in its functional (folded) state.

**FIGURE 4:**
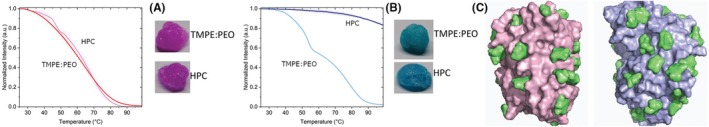
Modulated scanning fluorometry curves (non‐reversibility folding temperatures, *T*_nr_) for mCherry (A) and smURFP (B) in TMPE: PEO (light) and HPC (dark) coatings. (C) Docking of glucose units (green) with mCherry, left, and smURFP, right. Figure modified from ref. [20].

In the subgroup of Dr. Fuenzalida‐Werner within the chair for Biogenic Functional Materials, led by Professor Dr. Ruben Costa, the research focused on stabilizing FPs for optoelectronic applications. Therefore, a strategy similar to that previously described for MBP317‐347 was adopted, employing smURFP, a fluorescent protein of prokaryotic origin belonging to the phycobiliprotein family. smURFP was effectively stabilized in the solid state through favorable interactions with a biogenic hydroxypropyl cellulose (HPC) matrix, which locked the protein into a functional (folded) conformation.^20^ In contrast, the synthetic trimethylolpropane ethoxylate: polyethylene oxide composite (TMPE:PEO), whose PEO units are known to exert a denaturating effect, led to less rigid surroundings,^21^ distortion of smURFP's protein structure, and decreased thermal stability (Figure 4A). The rigid polysaccharide environment provided by HPC and the favorable interactions limit protein motion and photobleaching, which, despite a slight loss in fluorescence quantum yield, preserve smURFP (Figure 4B). Computational studies indicate that smURFP interacts strongly with cellulose through hydrogen bonding, engaging significantly more atoms than a control FP such as mCherry (namely, 147 vs. 111) resulting in higher total interaction energy (−598.8 kcal mol^−1^). For instance, the complexation of mCherry with HPC leads to slight destabilization (Figure 4C). The results were further validated by a computational methodology studying the effect of saccharides as crowders and stabilizers of eGFP.^22^ Consequently, supramolecular interactions with polysaccharides can be a good alternative for improving protein stability by locking the protein in its folded, functional state, as shown for MBP317‐347 and smURFP, being this, however, protein‐dependent.

The aforementioned strategy effectively parallels the mechanism of evolution to stabilize proteins in extreme environments, improving their kinetic stability, by using supramolecular interactions to lock proteins in a functional folded state.^23^, ^24^, ^25^ In detail, protein stability can be described as a complex phenomenon driven by thermodynamic and kinetic components. Traditionally, proteins are understood to exist in an equilibrium between their folded (functional) state and their unfolded state, and the energy difference between them measures their thermodynamic stability.^26^ Some proteins, however, are “locked” in their folded shape because unfolding them requires significant energy. This energy barrier between both states makes them unfold very slowly, even if unfolding would be favorable. This is the definition of kinetically stable proteins.^23^, ^26^, ^27^ Since unfolded proteins are more prone to interactions with other chemicals or themselves, the kinetic barrier is crucial for their susceptibility to stress conditions and for the functionality in extreme environments, like in the presence of synthetic polymers.^26^

Following this lead, evolution again offered inspiration. Kinetically stable proteins share few common structural patterns, one of which is their tendency to form multimers.^24^, ^26^, ^27^ There is often a correlation between kinetic stability and higher degrees of oligomerization, as it is hypothesized that monomeric proteins cannot achieve the level of topological complexity required for kinetic stability.^24^

However, this presents a challenge when working with FPs such as eGFP. In fact, despite their inherent oligomeric nature, the engineering of those proteins into bright and functional imaging tools is mainly accompanied by their monomerization. Consequently, these proteins cannot be easily engineered for increased stability, for example, by being made multimeric again because their structures are highly defined. Every non‐covalent interaction within the protein contributes to critical properties such as quantum yield, absorption coefficient, and excited‐state lifetime. Even minor mutations can drastically impair these functional parameters. This dilemma is common in protein engineering, and it is widely recognized as the stability–functionality trade‐off.^25^, ^28^

**FIGURE 5:**
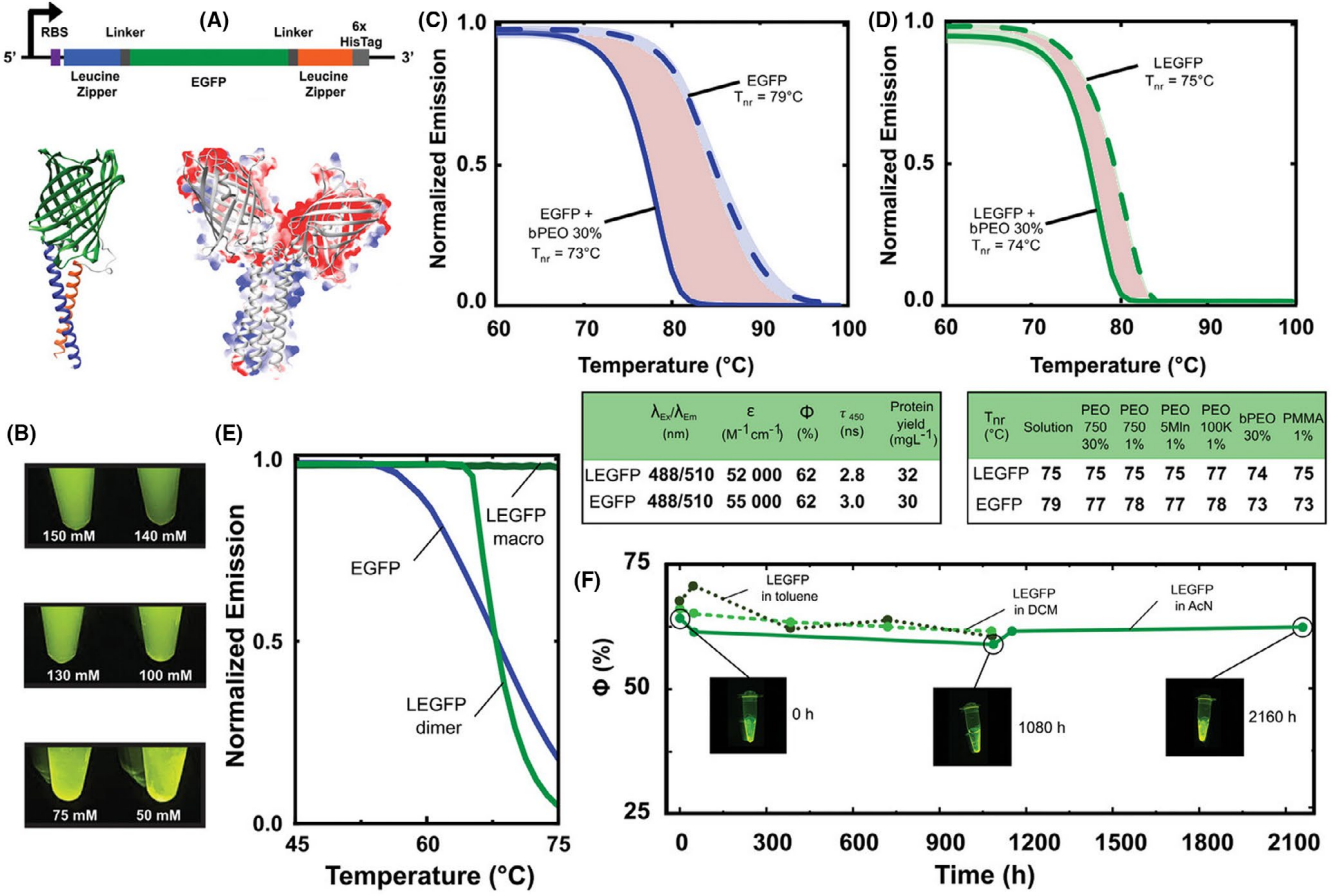
(A) LEGFP genetic construct, Alphafold2 model of monomeric LEGFP; the leucine zipper region is indicated in blue (N‐terminal) and orange (C‐terminal), and Superposition of LEGFP dimer (gray ribbons) with its columbic surface coloring. (B) LEGFP solutions showing oligomerization at different ionic strengths under blue light (450 nm) illumination. (C, D) Non‐reversibility folding temperatures (*T*_nr_) of EGFP and LEGFP in the presence and absence of bPEO 30%, an increase in red shade area translates to a less kinetically stable protein (E) *T*_nr_ of LEGFP macro‐oligomers, dimers, and EGFP in 95% AcN. (F) Fluorescent quantum yield over time of LEGFP macro‐oligomers in pure acetonitrile, dichloromethane, and toluene. Tables show the main photophysical properties and production yields of LEGFP and EGFP, as well as an overview of the non‐reversibility folding temperatures of LEGFP and EGFP in the presence and absence of synthetic polymers. Figure modified from ref. [29].

In Dr. Fuenzalida‐Werner's subgroup within the chair of Biogenic Functional Materials, an engineering strategy was devised to overcome protein stability issues in non‐biological environments such as in the presence of synthetic polymers or organic solvents, by using genetically encoded macro‐oligomerization. This approach stabilizes proteins without modifying eGFP itself; instead, it employs the fusion of complementary leucine zipper motifs at the N‐ and C‐terminal ends of proteins, creating a “gift bow” configuration (Figure 5A). These engineered constructs, termed LEGFP (leucine zipper fused to eGFP), facilitate strong protein–protein interactions primarily through electrostatic forces, enabling controlled oligomer formation modulated by ionic strength adjustments.^29^

This strategy maintained protein solubility during purification, effectively preventing the formation of inclusion bodies with a higher percentage of unfolded protein. Additionally, it allowed precise control over the onset of the oligomerization (Figure 5B). The resulting soluble constructs maintained the same photophysical properties as unmodified eGFP. Notably, the engineered proteins did not exhibit significantly enhanced thermodynamic stability; in fact, they showed slight reductions in melting temperature (*T*_m_) and the temperature of non‐reversibility (*T*_nr_) compared to their unmodified counterparts. However, in the presence of synthetic polymers such as polymethyl methacrylate (PMMA) and polyethylene oxide (PEO), LEGFP macro‐oligomers did not exhibit a drastic reduction in *T*_nr_, unlike unmodified proteins, which experienced a substantial loss of stability under the same conditions, as evidenced by a red shade area between both conditions in Figure 5C,D. Adjusting leucine zipper length effectively controlled macro‐oligomer sizes. Shorter leucine zippers produced smaller oligomers, consequently reducing *T*_nr_ in the presence of identical synthetic polymers. These experiments indicate that this strategy effectively increases the kinetic stability of eGFP without sacrificing its photophysical properties and indirectly proves that oligomers of a higher degree are more prone to being kinetically stable. The versatility of the genetically encoded oligomerization concept to other FPs was also demonstrated by fusing leucine zippers in a gift bow fashion to the N‐ and C‐termini of mNeptune2.5 (LmNeptune2.5), which is not phylogenetically close to eGFP. Like eGFP, the leucine zippers enable mNeptune2.5 to form macro‐oligomers controlled by ionic strength, without affecting the protein's yield of production and emissive properties, while increasing its resistance under harsh conditions.

Practically, macro‐oligomers exhibited markedly enhanced resilience, maintaining functional properties such as fluorescence for several months under harsh conditions, including exposure to pure organic solvents (acetonitrile, toluene, and dichloromethane) and within synthetic polymer environments (Figure 5E,F). This enhanced stability enables their use in materials as sustainable colorants, fluorescent materials, or other applications that require stable color sources. Significantly, as demonstrated in studies using different FPs, reducing protein backbone mobility, or “breathing” capacity, along with limiting oxygen accessibility to the chromophore, enhances photostability.^30^, ^31^ Thus, materials incorporating LEGFP macro‐oligomers showed notably improved photostability, resulting from restricted protein mobility and reduced heat generation within the polymeric structure embedded with oligomerized LEGFP. This effectively minimizes photo‐induced degradation typically observed in proteins under intense irradiation.

The obvious question is: why not generate complexes with increased kinetic stability by charge complementarity through electrostatic self‐assembly between oppositely charged proteins? Electrostatic self‐assembly offers numerous examples of controlled aggregation and the potential to provide on‐demand shapes, making it an ideal method for stabilizing proteins.^32^, ^33^ Unfortunately, most FPs available today—and most naturally occurring proteins—are not highly positively or negatively supercharged.^34^, ^35^ Generally, they exhibit only weak partial charges. In the case of FPs, nearly all that have evolved as labeling tools are either negatively charged or very close to charge neutrality.

**FIGURE 6:**
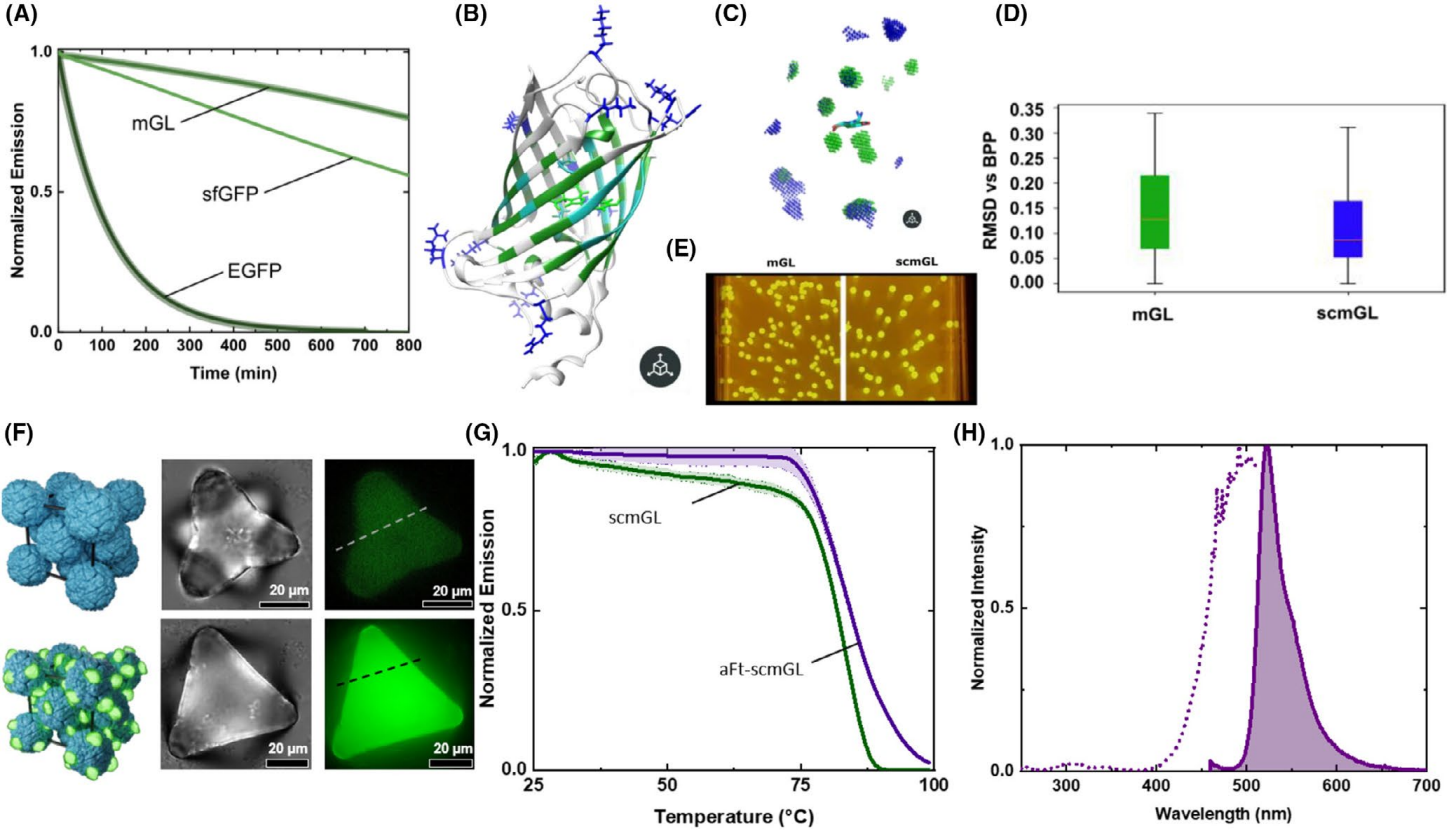
(A) Fluorescence over time at 70°C of mGL, sfGFP, and EGFP in PBS buffer solution. (B) Structural representation of scmGL; blue indicates the positively charged mutated amino acids, while dark green indicates the exclusion zone near the chromophore, with light blue marking the amino acids in direct contact. (C) Root‐mean‐square deviation (RMSD) analysis of Rosetta minimized populations of mGL and scmGL. (D) Comparison of the internal and peripheral volumes of mGL (green) and scmGL (blue). (E) *E. coli* colonies bearing mGL (left) and scmGL (right) expressing plasmids. (F) Comparison of aFt crystals and aFt‐scmGL crystals. Top lane: Bright‐field, fluorescence, and composite images of the aFt crystals formed using CdSO4 [50 mM]. Bottom line: Bright‐field, fluorescence, and composite images of aFt‐scmGL crystals with 50 mM NaCl. (G) *T*_nr_ of scmGL and aFt‐scmGL cocrystals. (H) Excitation and emission spectra of aFt‐scmGL cocrystals (measured at emission of 520 nm and excitation of 450 nm, respectively). Figure modified from ref. [36].

To address this limitation, a highly positively charged FP was developed in the subgroup of Dr. Fuenzalida‐Werner, aiming to enable interaction with naturally negatively charged proteins.^36^ However, creating highly positively supercharged FPs poses a significant challenge. Extensive surface mutations, typically necessary for supercharging, often destabilize protein folding and impair fluorescence. To overcome this, the most thermodynamically and kinetically stable FP—measured in terms of *T*_m_ and functionality over time at 70°C—among eGFP, sfGFP, and mGreenLantern (mGL) (Figure 6A) was identified. Ultimately, mGL was selected.

Recognizing that brightness loss in supercharged fluorescent proteins commonly results from increased chromophore flexibility and expanded internal space, structural disruptions near the chromophore were strategically minimized. By selecting polar, solvent‐exposed residues that do not interact with critical structural elements or salt bridges, 11 targeted mutations were selected to achieve a theoretical positive charge of +22, while carefully preserving chromophore packing and conformational stability (Figure 6B,C).

Computational modeling confirmed that the mutations did not increase the internal space surrounding the chromophore or significantly affected protein flexibility (Figure 6D). Experimentally, the resulting supercharged mGL, scmGL, demonstrated slightly reduced folding reversibility (*T*_nr_ area) but retained high thermostability (*T*_m_), fluorescence intensity, quantum yield, and broad pH compatibility, comparable to its parental protein, mGL (Figure 6E). Thus, the tailored mutation strategy successfully produced a positively supercharged protein with minimal compromise to its structural and photophysical properties.

Next, in collaboration with researchers from the group of Professor Dr. Mauri A. Kostiainen and Dr. Eduardo Anaya, the successful electrostatic self‐assembly of aFt (apoferritin) protein cages with scmGL was demonstrated, highlighting the improved stability of the resulting complexes (Figure 6F,G). Using dynamic light scattering (DLS), a concentration‐dependent increase in scattering intensity and hydrodynamic diameter was observed, indicating the formation of large aFt–scmGL complexes.^36^ The assembly was reversible and disassembled when the ionic strength was increased, confirming that electrostatic interactions are the primary driving force. Control experiments with negatively charged mGL failed to yield cocrystals, further supporting the critical role of scmGL's localized positive charges. Optically, UV–vis and fluorescence studies revealed that the emission spectrum remained similar to that of scmGL and mGL in solution, with a fluorescence quantum yield of about 70%. A broader excitation spectrum was observed, likely due to slight changes in chromophores resulting from protein aggregation (Figure 6H). This aggregation also caused a decrease in the excited‐state lifetime, which is attributable to the higher refractive index in the multimeric environment. The thermodynamic properties of aFt–scmGL crystals were then evaluated.^36^ The crystals showed improved stability, with the *T*_nr_ area increasing from 52.8 to 56.3 and the melting temperature *T*_m_ rising from 63 to 72°C, exceeding that of the parent mGL protein (Figure 6G). More importantly, the assemblies allowed the protein to be fully functional in the extreme environment of pure silicone polymer with no reduction in fluorescence quantum yield. This demonstrates that, similar to other biopolymers, proteins can interface with silicone.^37^, ^38^ In this regard, a recent work shows that sugars can be a promising interface not only between biopolymers and synthetic polymers but also between microorganisms and synthetic polymers^39^.

## CAN FLUORESCENT PROTEINS BE USED IN OTHER APPLICATIONS?

FPs exhibit complex photophysics, enabling not only fluorescent imaging but also advanced applications such as super‐resolution microscopy via photoswitchable proteins, fluorescence lifetime imaging, and optoacoustic imaging.^40^, ^41^, ^42^ Despite this extensive range of uses, circularly polarized luminescence (CPL) emission has remained unexplored in FPs.

While circular dichroism (CD) involves the differential absorption of left and right‐handed circularly polarized light by molecules with chiral ground electronic states, CPL refers to the differential emission of circularly polarized light from molecules possessing a chiral excited state.^43^ Typically, CPL signals are weaker than the already weak CD signals.

Historically, efforts in designing chiroptical materials (materials capable of emitting CPL) have predominantly relied on small organic molecules (e.g., ketones, cyclophanes, BODIPYs, helicenes, pyrene intramolecular excimers) and metal complexes (d‐metal and rare‐earth compounds).^44^ These approaches often sacrifice molar absorptivity, processability, and sustainability to achieve high dissymmetry factors (*g*_lum_). Over 90% of published chiroptical material families report average CPL brightness (*B*_CPL_) values below 150 M^−1^ cm^−1^. This relatively low performance arises from strategies compromising fluorescence quantum yield or molecular absorptivity in pursuit of large *g*_lum_ values.^44^ However, Nagata et al. demonstrated that available experimental data do not fully support the inverse relationship between fluorescence quantum yield and CPL intensity.^45^

Considering this background, fluorescent proteins began to be investigated as potential biogenic CPL emitters in the subgroup of Dr. Fuenzalida‐Werner, motivated by the concept that chiral selectivity at the supramolecular level is favored by natural evolution. Consequently, FPs could represent ideal natural emitters, potentially offering high CPL brightness across visible and near‐infrared spectra.^46^

**FIGURE 7:**
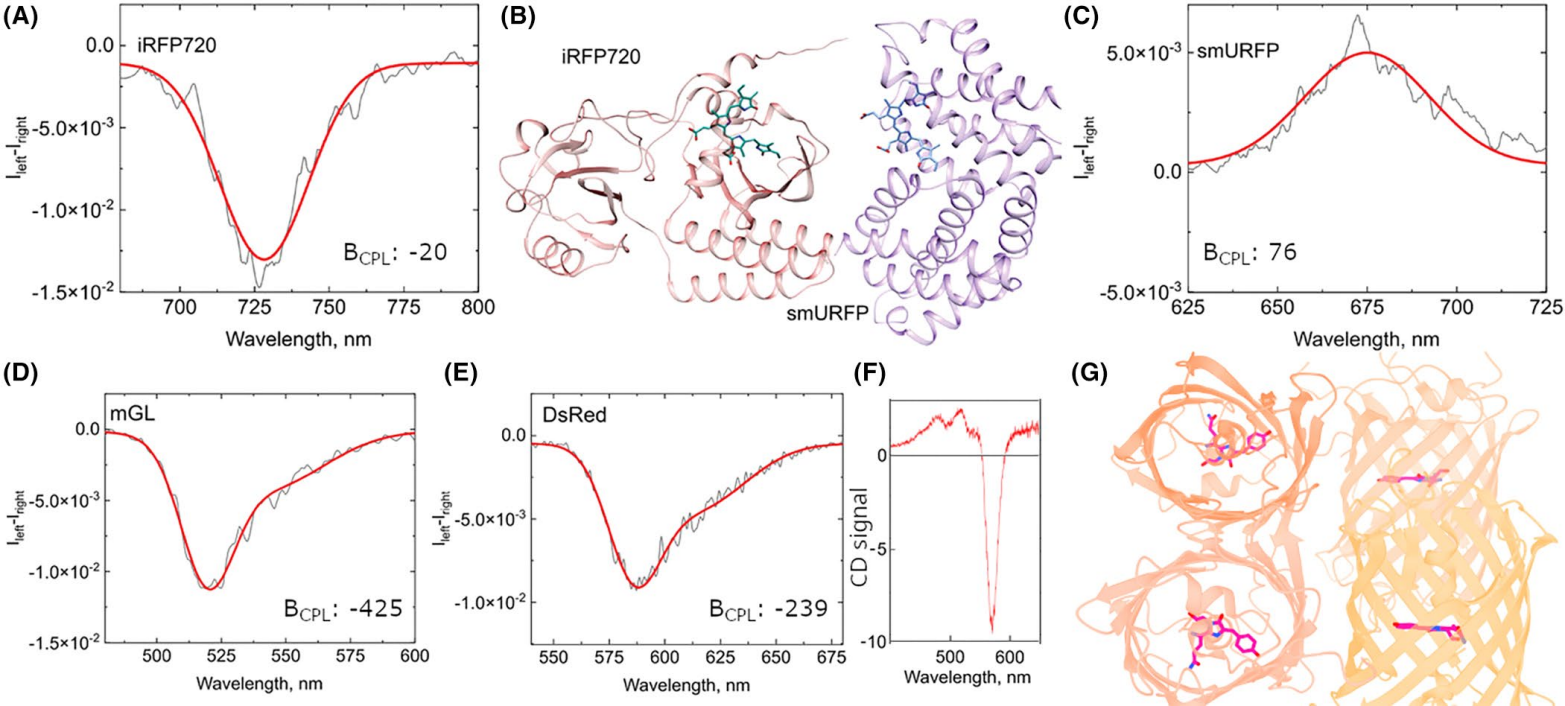
(A) CPL signal for IRFP720 (B) Comparison between the locked conformation of BV bound to smURFP (PDB: 6FZN^33^) and iRFP720 (Model according to PDB: 8AFK). (C) CPL signal for smURFP (D, E) CPL signal for mGL and DsRed, respectively. (F) Circular Dichroism spectra for DsRed. (G) Structure of DsRed. Figure modified from ref. [46].

The analysis covered most known FPs classes, examining ground‐state chirality, steady‐state, and time‐resolved fluorescence properties. Notably, ligand‐based FPs such as UnaG, smURFP, and iRFP720, which utilize natural chromophores bilirubin (BR) and biliverdin (BV), displayed substantial CPL signals. These proteins exhibited *g*_lum_ values ranging from 5.0 × 10^−3^ to −1.3 × 10^−2^, with *B*_CPL_ reaching up to −246 M^−1^ cm^−1^. The CPL emission in these cases was significantly influenced by structural factors, including chromophore conformational constraints within the protein scaffold and the chiral environment provided by nearby amino acids (Figure 7A–C). For example, smURFP showed a positive CPL signal correlated with its CD signal. At the same time, iRFP exhibited a negative CPL signal correlated with its CD spectrum (Figure 7A–C), making both among the most red‐shifted CPL emitters reported.

β‐barrel‐structured FPs like eGFP, sfGFP, and mGL exhibited exceptionally high CPL performances. Among these, mGL stood out, achieving an exceptional *B*_CPL_ value of −425 M^−1^ cm^−1^, attributed to its highly rigid chromophore structure, favorable environmental polarity within the protein pocket, and a pronounced cis chromophore conformation (Figure 7D). Similarly, red‐emitting proteins, including DsRed, dTomato, and mCherry, demonstrated significant *B*_CPL_ values, with DsRed reaching a notable *B*_CPL_ of −239 M^−1^ cm^−1^. This prominent CPL activity was explained through exciton coupling, observable in the CD spectra of DsRed (Figure 7E–G), resulting from its tetrameric structure, close chromophore distances, structural rigidity, and the environment of the chromophore‐binding cavity (Figure 7G).

Structural analyses supported these findings, revealing that chromophore rigidity, the surrounding amino acid environment, supramolecular packing, and exciton coupling are critical for understanding CPL emission capacity in FPs. This knowledge opens up avenues for genetically tuning CPL values in future applications.

Practically, the best‐performing red and green CPL emitters identified in this study—mGL and DsRed—were tested as potential chiroptical materials by embedding them into hydroxylpropyl cellulose (HPC) polymer matrices. Although the stability of these β‐barrel proteins, similar to mCherry as described previously, was somewhat affected by HPC, the polymer is a widely used matrix for stabilizing CPL emitters. CPL intensity remained well preserved within these polymer coatings at lower protein concentrations. However, increased protein concentration led to aggregation, reducing fluorescence efficiency and CPL performance. Nevertheless, the polymer coatings successfully maintained stable polarization characteristics, even after prolonged exposure to light.

## FUTURE PERSPECTIVE

Integrating proteins into materials science requires a cohesive approach that combines advanced protein engineering strategies to enhance kinetic stability with innovative materials science methodologies focused on developing compounds, such as polymers or additives, that can effectively stabilize proteins. Historically, these two domains have evolved independently; however, significant cross‐disciplinary collaboration is now essential to make substantial advances in the field.

For instance, in the field of bio‐inspired CPL materials, there is a pressing need for new biodegradable polymers that preserve the intrinsic optical activity of protein‐based emitters while minimizing scattering. Developing such polymer matrices will enable broader applications by maintaining optical properties and supporting environmental sustainability.

Moreover, although fluorescent proteins already find diverse applications in material science, the broader integration of various protein classes, such as enzymes, into practical materials demands a deeper consideration of protein dynamics and their correlation with long‐term functionality. To address this, leveraging cutting‐edge machine learning techniques could be pivotal. Specifically, machine learning models could rapidly predict and optimize protein–material interactions, accelerating the design and deployment of robust protein‐based materials. Consequently, a targeted exploration of computational methods to fine‐tune interactions between proteins and embedding materials should become a cornerstone strategy for future advancements in protein‐integrated materials science.

## AUTHOR CONTRIBUTIONS

Prof. Dr. J. P. Fuenzalida‐Werner and Dr. Mattia Nieddu share corresponding authorship of this review.

## FUNDING INFORMATION

This review was carried out on the framework of the RYC2023‐042520‐I funded by MICIU/AEI/10.13039/501100011033 and by “FSE+”.

## Data Availability

Data sharing is not applicable to this article as no new data were created or analyzed in this study.
